# Redistributing ill-defined deaths in the German Burden of Disease study BURDEN 2020: Annelene Wengler

**DOI:** 10.1093/eurpub/ckac129.621

**Published:** 2022-10-25

**Authors:** A Wengler, E von der Lippe

**Affiliations:** Epidemiology and Health Monitoring, Robert Koch Institute, Berlin, Germany

## Abstract

Having valid information on mortality within a country is of great importance for public health planning. This includes knowing the causes of death (CoD) within a population. However, these data are not always suitable for Burden of Disease calculations from the start and hence, need some realignment in advance. The CoD statistics in Germany include a relatively high share (26% in 2017) of ill-defined deaths (IDD) - using the definition of the Global Burden of Disease Study. Additionally, only the underlying CoD is included in the national statistics and no multicausal data are available yet. As part of the German Burden of Disease project BURDEN 2020 we redistributed IDD to valid CoD using a process of proportional redistribution. To do so, we made use of the distribution of the valid ICD-codes in the data. In the proposed presentation, we use examples of stroke, diabetes, and heart failure to illustrate how IDD were reallocated. After redistribution, the largest increases for both women and men were seen for lower respiratory infections, diabetes mellitus, and stroke. The numbers of deaths for these causes more than doubled after redistribution. Within this project, we carried out the first comprehensive redistribution of IDD for German CoD statistics.

